# 4-{2-[(5-Bromo-2-hydroxy­benzyl­idene)amino]eth­yl}benzene­sulfonamide

**DOI:** 10.1107/S1600536809036083

**Published:** 2009-09-12

**Authors:** Zahid H. Chohan, Hazoor A. Shad, M. Nawaz Tahir, Khalid H. Thebo

**Affiliations:** a Department of Chemistry, Bahauddin Zakariya University, Multan 60800, Pakistan; b Department of Physics, University of Sargodha, Sargodha, Pakistan; c School of Chemistry, University of Manchester, Oxford Road, Manchester M13 9PL, England

## Abstract

In the title compound, C_15_H_15_BrN_2_O_3_S, the dihedral angle between the benzene rings is 6.1 (2)° and an intra­molecular O—H⋯N hydrogen bond helps to establish the conformation. In the crystal structure, the mol­ecules are linked by way of N—H⋯O and C—H⋯O hydrogen bonds.

## Related literature

For related structures, see: Chohan *et al.* (2008 ▶, 2009 ▶). For graph-set notation, see: Bernstein *et al.* (1995 ▶).
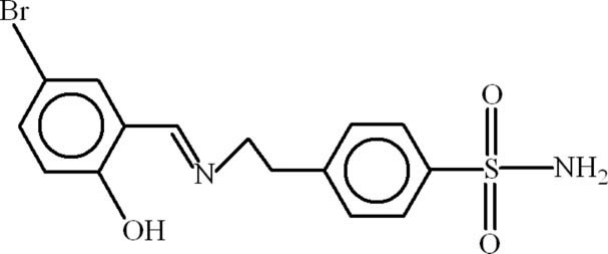


## Experimental

### 

#### Crystal data

C_15_H_15_BrN_2_O_3_S
*M* *_r_* = 383.26
Orthorhombic, 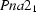

*a* = 50.544 (6) Å
*b* = 6.3146 (10) Å
*c* = 4.8625 (5) Å
*V* = 1551.9 (3) Å^3^
*Z* = 4
Mo *K*α radiation
μ = 2.80 mm^−1^
*T* = 296 K
0.28 × 0.14 × 0.12 mm

#### Data collection

Bruker Kappa APEXII CCD diffractometer
Absorption correction: multi-scan (*SADABS*; Bruker, 2005 ▶) *T*_min_ = 0.633, *T*_max_ = 0.714
8263 measured reflections
2844 independent reflections
2523 reflections with *I* > 2σ(*I*)
*R*_int_ = 0.035

#### Refinement

*R*[*F* ^2^ > 2σ(*F* ^2^)] = 0.048
*wR*(*F* ^2^) = 0.100
*S* = 1.12
2844 reflections
213 parameters
1 restraint
H atoms treated by a mixture of independent and constrained refinement
Δρ_max_ = 0.41 e Å^−3^
Δρ_min_ = −0.79 e Å^−3^
Absolute structure: Flack (1983 ▶), 1212 Friedel pairs
Flack parameter: 0.050 (15)

### 

Data collection: *APEX2* (Bruker, 2007 ▶); cell refinement: *SAINT* (Bruker, 2007 ▶); data reduction: *SAINT*; program(s) used to solve structure: *SHELXS97* (Sheldrick, 2008 ▶); program(s) used to refine structure: *SHELXL97* (Sheldrick, 2008 ▶); molecular graphics: *ORTEP-3 for Windows* (Farrugia, 1997 ▶) and *PLATON* (Spek, 2009 ▶); software used to prepare material for publication: *WinGX* (Farrugia, 1999 ▶) and *PLATON*.

## Supplementary Material

Crystal structure: contains datablocks global, I. DOI: 10.1107/S1600536809036083/hb5091sup1.cif

Structure factors: contains datablocks I. DOI: 10.1107/S1600536809036083/hb5091Isup2.hkl

Additional supplementary materials:  crystallographic information; 3D view; checkCIF report

## Figures and Tables

**Table 1:** Hydrogen-bond geometry (Å, °)

*D*—H⋯*A*	*D*—H	H⋯*A*	*D*⋯*A*	*D*—H⋯*A*
O1—H1*O*⋯N1	0.82	1.88	2.602 (6)	147
N2—H2*A*⋯O3^i^	0.80 (5)	2.30 (5)	3.076 (6)	163 (5)
C15—H15⋯O2^ii^	0.93	2.49	3.259 (7)	140

Symmetry codes: (i) 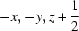; (ii) 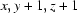.

**Fig. 1.**
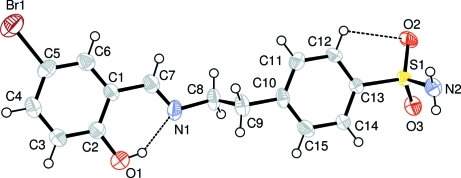
View of (I) with displacement ellipsoids drawn at the 50% probability level. H atoms are shown by small circles of arbitrary radii. The dotted line indicate the intramolecular H-bond.

**Fig. 2.**
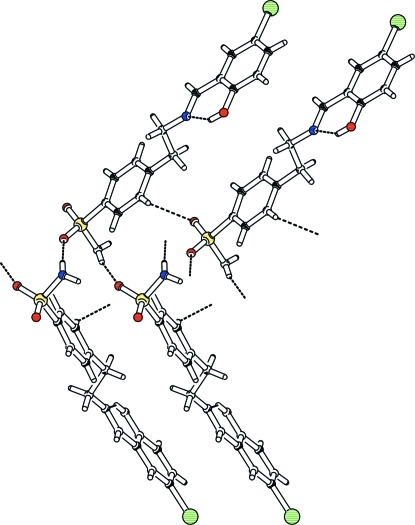
The partial packing (PLATON; Spek, 2009) which shows that molecules form polymeric chains.

## supplementary crystallographic information

### Comment

As part of our ongoing studies of sulfonamide derivatives (Chohan *et al.* 2009), we now report the preparation and crystal structure of title compound (I), (Fig. 1).

The crystal structure of (II) 4-{2-[(5-chloro-2 hydroxybenzylidene)amino]ethyl} -benzenesulfonamide (Chohan *et al.*, 2008) has been reported which differs from (I) due to chloro substitution instead of bromo.

In (I), the benzene rings A (C1–C6) of 5-bromosalicylaldehyde and B (C10–C15) of sulfanilamide moiety are oriented at a dihedral angle of 6.1 (3)° whereas its value as observed in (II) is 23.95 (18)°. The Br1 and S1 atoms are at a distance of -0.024 (7) and -0.167 (6) Å from the mean square planes of rings A and B, respectively. There exist two intramolecular H-bondings (Table 1, Fig. 1) forming S(5) and S(6) ring motifs (Bernstein *et al.*, 1995). Two intermolecular H-bondings (Table 1, Fig. 2) link the molecules in infinite one dimensional polymeric network extending along the *c* axis.

### Experimental

An ethanol solution (20 ml) of 4-(2-aminoethyl) benzene sulfonamide (0.4005 g, 2 mmol) was added to an ethanol solution (10 ml) of 5-bromosalicylaldehyde (0.402 g, 2 mmol). The reaction mixture was refluxed for 3 h. The colour of the solution gradually changed from colourless to greenish yellow. The solution was cooled to room temperature, filtered and the volume was reduced to about one-third on the rotary evaporator. The resulting mixture was allowed to stand for 10 days, after which bright yellow needles of (I) were obtained.

### Refinement

The coordinates of H atoms of NH_2_ group were refined. The other H atoms were positioned geometrically with O—H = 0.82, C—H = 0.93 and 0.97 Å for aromatic and methylene H atoms and constrained to ride on their parent atoms, with *U*_iso_(H) = xUeq(C, N, O), where *x* = 1.2 for all H atoms.

### Crystal data

**Table:**

C_15_H_15_BrN_2_O_3_S	*F*(000) = 776
*M**_r_* = 383.26	*D*_x_ = 1.640 Mg m^−^^3^
Orthorhombic, *P**n**a*2_1_	Mo *K*α radiation, λ = 0.71073 Å
Hall symbol: P 2c -2n	Cell parameters from 2932 reflections
*a* = 50.544 (6) Å	θ = 2.4–28.3°
*b* = 6.3146 (10) Å	µ = 2.80 mm^−^^1^
*c* = 4.8625 (5) Å	*T* = 296 K
*V* = 1551.9 (3) Å^3^	Needle, yellow
*Z* = 4	0.28 × 0.14 × 0.12 mm

### Data collection

**Table:**

Bruker Kappa APEXII CCD diffractometer	2844 independent reflections
Radiation source: fine-focus sealed tube	2523 reflections with *I* > 2σ(*I*)
graphite	*R*_int_ = 0.035
Detector resolution: 7.80 pixels mm^-1^	θ_max_ = 25.5°, θ_min_ = 2.4°
ω scans	*h* = −52→60
Absorption correction: multi-scan (*SADABS*; Bruker, 2005)	*k* = −7→6
*T*_min_ = 0.633, *T*_max_ = 0.714	*l* = −5→5
8263 measured reflections	

### Refinement

**Table:**

Refinement on *F*^2^	Secondary atom site location: difference Fourier map
Least-squares matrix: full	Hydrogen site location: inferred from neighbouring sites
*R*[*F*^2^ > 2σ(*F*^2^)] = 0.048	H atoms treated by a mixture of independent and constrained refinement
*wR*(*F*^2^) = 0.100	*w* = 1/[σ^2^(*F*_o_^2^) + 2.7358*P*] where *P* = (*F*_o_^2^ + 2*F*_c_^2^)/3
*S* = 1.12	(Δ/σ)_max_ = 0.001
2844 reflections	Δρ_max_ = 0.41 e Å^−^^3^
213 parameters	Δρ_min_ = −0.79 e Å^−^^3^
1 restraint	Absolute structure: Flack (1983), 1212 Friedel pairs
Primary atom site location: structure-invariant direct methods	Flack parameter: 0.050 (15)

### Special details

**Table:**

Geometry. Bond distances, angles *etc*. have been calculated using the rounded fractional coordinates. All su's are estimated from the variances of the (full) variance-covariance matrix. The cell e.s.d.'s are taken into account in the estimation of distances, angles and torsion angles
Refinement. Refinement of *F*^2^ against ALL reflections. The weighted *R*-factor *wR* and goodness of fit *S* are based on *F*^2^, conventional *R*-factors *R* are based on *F*, with *F* set to zero for negative *F*^2^. The threshold expression of *F*^2^ > σ(*F*^2^) is used only for calculating *R*-factors(gt) *etc*. and is not relevant to the choice of reflections for refinement. *R*-factors based on *F*^2^ are statistically about twice as large as those based on *F*, and *R*- factors based on ALL data will be even larger.

### Fractional atomic coordinates and isotropic or equivalent isotropic displacement parameters (Å^2^)

**Table:**

	*x*	*y*	*z*	*U*_iso_*/*U*_eq_	
Br1	0.24179 (1)	0.37198 (12)	1.16449 (17)	0.0633 (2)	
S1	0.03204 (2)	−0.2434 (2)	−0.5033 (3)	0.0276 (3)	
O1	0.15029 (7)	0.8114 (6)	0.6129 (10)	0.0543 (16)	
O2	0.04542 (7)	−0.4313 (6)	−0.5921 (8)	0.0460 (12)	
O3	0.02060 (7)	−0.1032 (6)	−0.7008 (7)	0.0398 (11)	
N1	0.14244 (8)	0.4653 (8)	0.3341 (10)	0.0420 (16)	
N2	0.00760 (8)	−0.3200 (7)	−0.3108 (12)	0.0367 (14)	
C1	0.17777 (8)	0.5013 (8)	0.6554 (13)	0.0330 (14)	
C2	0.17084 (9)	0.7071 (9)	0.7344 (10)	0.0360 (17)	
C3	0.18524 (10)	0.8111 (9)	0.9375 (12)	0.0437 (19)	
C4	0.20599 (10)	0.7116 (9)	1.0649 (10)	0.0403 (19)	
C5	0.21316 (9)	0.5108 (9)	0.9871 (12)	0.0373 (16)	
C6	0.19906 (10)	0.4058 (9)	0.7859 (11)	0.0397 (17)	
C7	0.16276 (10)	0.3869 (9)	0.4471 (11)	0.0390 (17)	
C8	0.12783 (10)	0.3344 (9)	0.1349 (13)	0.0463 (19)	
C9	0.10328 (11)	0.2403 (10)	0.2692 (11)	0.0450 (19)	
C10	0.08639 (10)	0.1205 (9)	0.0700 (9)	0.0330 (16)	
C11	0.09253 (10)	−0.0829 (9)	−0.0103 (12)	0.0420 (19)	
C12	0.07651 (10)	−0.1935 (8)	−0.1920 (12)	0.0380 (17)	
C13	0.05431 (8)	−0.0959 (7)	−0.2953 (9)	0.0263 (16)	
C14	0.04792 (10)	0.1065 (8)	−0.2252 (10)	0.0323 (17)	
C15	0.06412 (10)	0.2172 (9)	−0.0426 (12)	0.0403 (17)	
H1O	0.14259	0.73100	0.50732	0.0815*	
H2A	−0.0025 (11)	−0.224 (8)	−0.292 (14)	0.0439*	
H2B	0.0123 (10)	−0.405 (9)	−0.181 (13)	0.0439*	
H3	0.18077	0.94875	0.98695	0.0522*	
H4	0.21522	0.78030	1.20423	0.0481*	
H6	0.20392	0.26884	0.73703	0.042 (16)*	
H7	0.16828	0.25207	0.39564	0.06 (2)*	
H8A	0.13916	0.22125	0.06878	0.0554*	
H8B	0.12268	0.42041	−0.02147	0.0554*	
H9A	0.10868	0.14594	0.41624	0.0540*	
H9B	0.09291	0.35359	0.35006	0.0540*	
H11	0.10770	−0.14723	0.05852	0.06 (2)*	
H12	0.08071	−0.33135	−0.24312	0.030 (14)*	
H14	0.03291	0.17034	−0.29820	0.040 (14)*	
H15	0.06003	0.35623	0.00408	0.030 (13)*	

### Atomic displacement parameters (Å^2^)

**Table:**

	*U*^11^	*U*^22^	*U*^33^	*U*^12^	*U*^13^	*U*^23^
Br1	0.0515 (3)	0.0879 (5)	0.0505 (3)	0.0203 (3)	−0.0093 (4)	−0.0043 (4)
S1	0.0311 (6)	0.0282 (6)	0.0234 (5)	−0.0029 (5)	0.0027 (5)	−0.0059 (5)
O1	0.045 (2)	0.047 (3)	0.071 (3)	0.0065 (19)	−0.013 (2)	−0.008 (2)
O2	0.047 (2)	0.036 (2)	0.055 (2)	0.0026 (18)	0.0051 (19)	−0.0254 (19)
O3	0.047 (2)	0.042 (2)	0.0305 (18)	−0.0067 (19)	−0.0041 (16)	0.0036 (17)
N1	0.030 (2)	0.052 (3)	0.044 (3)	−0.010 (2)	−0.001 (2)	−0.009 (2)
N2	0.040 (2)	0.036 (3)	0.034 (2)	−0.0072 (19)	0.005 (3)	0.002 (2)
C1	0.028 (2)	0.034 (3)	0.037 (2)	−0.007 (2)	0.010 (3)	−0.009 (3)
C2	0.028 (3)	0.037 (3)	0.043 (3)	−0.004 (2)	0.008 (2)	−0.005 (2)
C3	0.042 (3)	0.036 (3)	0.053 (4)	−0.002 (2)	−0.002 (3)	−0.008 (3)
C4	0.040 (3)	0.046 (4)	0.035 (3)	−0.011 (3)	0.004 (2)	−0.009 (2)
C5	0.025 (2)	0.052 (3)	0.035 (3)	0.003 (2)	0.008 (2)	0.003 (3)
C6	0.038 (3)	0.037 (3)	0.044 (3)	−0.003 (3)	0.008 (2)	−0.008 (3)
C7	0.036 (3)	0.040 (3)	0.041 (3)	−0.006 (2)	0.007 (2)	−0.008 (3)
C8	0.042 (3)	0.057 (4)	0.040 (3)	−0.013 (3)	−0.011 (3)	−0.011 (3)
C9	0.046 (3)	0.051 (4)	0.038 (3)	−0.013 (3)	−0.005 (2)	−0.007 (3)
C10	0.031 (2)	0.041 (3)	0.027 (3)	−0.015 (2)	0.0053 (19)	−0.004 (2)
C11	0.034 (3)	0.047 (4)	0.045 (3)	0.003 (2)	−0.010 (3)	−0.004 (3)
C12	0.042 (3)	0.025 (3)	0.047 (3)	0.004 (2)	−0.007 (3)	−0.009 (3)
C13	0.031 (2)	0.028 (3)	0.020 (3)	−0.0034 (19)	0.004 (2)	−0.003 (2)
C14	0.030 (3)	0.028 (3)	0.039 (3)	−0.002 (2)	−0.002 (2)	−0.002 (2)
C15	0.045 (3)	0.034 (3)	0.042 (3)	−0.004 (2)	0.005 (3)	−0.011 (3)

### Geometric parameters (Å, °)

**Table:**

Br1—C5	1.899 (5)	C9—C10	1.496 (8)
S1—O2	1.432 (4)	C10—C15	1.393 (7)
S1—O3	1.428 (4)	C10—C11	1.378 (8)
S1—N2	1.624 (5)	C11—C12	1.387 (8)
S1—C13	1.777 (4)	C12—C13	1.375 (7)
O1—C2	1.364 (6)	C13—C14	1.362 (7)
O1—H1O	0.8200	C14—C15	1.396 (7)
N1—C7	1.266 (7)	C3—H3	0.9300
N1—C8	1.472 (8)	C4—H4	0.9300
N2—H2A	0.80 (5)	C6—H6	0.9300
N2—H2B	0.86 (6)	C7—H7	0.9300
C1—C7	1.457 (8)	C8—H8A	0.9700
C1—C2	1.400 (8)	C8—H8B	0.9700
C1—C6	1.387 (7)	C9—H9A	0.9700
C2—C3	1.392 (7)	C9—H9B	0.9700
C3—C4	1.371 (7)	C11—H11	0.9300
C4—C5	1.372 (8)	C12—H12	0.9300
C5—C6	1.380 (8)	C14—H14	0.9300
C8—C9	1.523 (8)	C15—H15	0.9300
			
Br1···C6^i^	3.720 (5)	C7···C5^vi^	3.479 (7)
Br1···C4^ii^	3.433 (5)	C8···C1^vi^	3.594 (8)
Br1···C5^ii^	3.584 (5)	C13···O3^i^	3.356 (6)
Br1···H4^iii^	3.1700	C14···O3^i^	3.188 (6)
O1···N1	2.602 (6)	C15···O2^x^	3.259 (7)
O2···C15^iv^	3.259 (7)	C15···O3^i^	3.420 (7)
O3···N2^v^	3.076 (6)	C7···H1O	2.4200
O3···C14^vi^	3.188 (6)	C11···H8A	3.0600
O3···C15^vi^	3.420 (7)	H1O···N1	1.8800
O3···C13^vi^	3.356 (6)	H1O···C7	2.4200
O2···H12	2.5400	H2A···O3^vii^	2.30 (5)
O2···H15^iv^	2.4900	H2B···N2^xi^	2.70 (6)
O2···H9B^iv^	2.7700	H4···Br1^xii^	3.1700
O3···H14	2.6800	H6···H7	2.4500
O3···H2A^v^	2.30 (5)	H7···H6	2.4500
O3···H14^v^	2.7800	H7···H8A	2.1700
N1···O1	2.602 (6)	H8A···C11	3.0600
N2···O3^vii^	3.076 (6)	H8A···H7	2.1700
N1···H1O	1.8800	H9A···H11	2.5400
N2···H2B^viii^	2.70 (6)	H9B···O2^x^	2.7700
C1···C4^vi^	3.470 (8)	H9B···H15	2.3600
C1···C8^i^	3.594 (8)	H11···H9A	2.5400
C4···C1^i^	3.470 (8)	H12···O2	2.5400
C4···C7^i^	3.526 (8)	H12···H15^xiii^	2.5400
C4···Br1^ix^	3.433 (5)	H14···O3	2.6800
C5···C7^i^	3.479 (7)	H14···O3^vii^	2.7800
C5···Br1^ix^	3.584 (5)	H15···O2^x^	2.4900
C6···Br1^vi^	3.720 (5)	H15···H9B	2.3600
C7···C4^vi^	3.526 (8)	H15···H12^xiv^	2.5400
			
O2—S1—O3	120.1 (2)	S1—C13—C12	119.3 (4)
O2—S1—N2	106.6 (2)	C12—C13—C14	121.5 (4)
O2—S1—C13	107.9 (2)	S1—C13—C14	119.0 (3)
O3—S1—N2	105.3 (2)	C13—C14—C15	119.4 (5)
O3—S1—C13	108.3 (2)	C10—C15—C14	120.3 (5)
N2—S1—C13	108.1 (2)	C2—C3—H3	120.00
C2—O1—H1O	109.00	C4—C3—H3	120.00
C7—N1—C8	118.2 (5)	C3—C4—H4	120.00
S1—N2—H2A	109 (4)	C5—C4—H4	120.00
S1—N2—H2B	114 (3)	C1—C6—H6	119.00
H2A—N2—H2B	124 (6)	C5—C6—H6	119.00
C2—C1—C7	121.4 (4)	N1—C7—H7	119.00
C6—C1—C7	120.4 (5)	C1—C7—H7	119.00
C2—C1—C6	118.2 (5)	N1—C8—H8A	110.00
O1—C2—C1	121.3 (5)	N1—C8—H8B	110.00
O1—C2—C3	118.5 (5)	C9—C8—H8A	110.00
C1—C2—C3	120.1 (5)	C9—C8—H8B	110.00
C2—C3—C4	120.3 (5)	H8A—C8—H8B	108.00
C3—C4—C5	120.1 (5)	C8—C9—H9A	109.00
Br1—C5—C4	120.2 (4)	C8—C9—H9B	109.00
Br1—C5—C6	119.6 (4)	C10—C9—H9A	109.00
C4—C5—C6	120.2 (5)	C10—C9—H9B	109.00
C1—C6—C5	121.1 (5)	H9A—C9—H9B	108.00
N1—C7—C1	122.0 (5)	C10—C11—H11	119.00
N1—C8—C9	110.2 (5)	C12—C11—H11	119.00
C8—C9—C10	112.6 (4)	C11—C12—H12	121.00
C9—C10—C15	119.6 (5)	C13—C12—H12	121.00
C11—C10—C15	118.6 (5)	C13—C14—H14	120.00
C9—C10—C11	121.8 (5)	C15—C14—H14	120.00
C10—C11—C12	121.2 (5)	C10—C15—H15	120.00
C11—C12—C13	118.9 (5)	C14—C15—H15	120.00
			
O2—S1—C13—C12	−16.3 (5)	C2—C3—C4—C5	1.9 (8)
O2—S1—C13—C14	168.7 (4)	C3—C4—C5—Br1	−179.7 (4)
O3—S1—C13—C12	−147.7 (4)	C3—C4—C5—C6	−1.9 (8)
O3—S1—C13—C14	37.2 (4)	Br1—C5—C6—C1	178.9 (4)
N2—S1—C13—C12	98.7 (4)	C4—C5—C6—C1	1.1 (8)
N2—S1—C13—C14	−76.4 (4)	N1—C8—C9—C10	175.1 (5)
C8—N1—C7—C1	−177.3 (5)	C8—C9—C10—C11	79.5 (7)
C7—N1—C8—C9	101.1 (6)	C8—C9—C10—C15	−99.3 (6)
C6—C1—C2—O1	179.3 (5)	C9—C10—C11—C12	179.0 (5)
C6—C1—C2—C3	0.4 (8)	C15—C10—C11—C12	−2.2 (8)
C7—C1—C2—O1	−2.2 (8)	C9—C10—C15—C14	−179.0 (5)
C7—C1—C2—C3	178.8 (5)	C11—C10—C15—C14	2.3 (8)
C2—C1—C6—C5	−0.4 (8)	C10—C11—C12—C13	0.8 (8)
C7—C1—C6—C5	−178.8 (5)	C11—C12—C13—S1	−174.3 (4)
C2—C1—C7—N1	−2.0 (8)	C11—C12—C13—C14	0.6 (8)
C6—C1—C7—N1	176.4 (5)	S1—C13—C14—C15	174.4 (4)
O1—C2—C3—C4	179.9 (5)	C12—C13—C14—C15	−0.5 (7)
C1—C2—C3—C4	−1.2 (8)	C13—C14—C15—C10	−1.0 (8)

Symmetry codes: (i) *x*, *y*, *z*+1; (ii) −*x*+1/2, *y*−1/2, *z*+1/2; (iii) −*x*+1/2, *y*−1/2, *z*−1/2; (iv) *x*, *y*−1, *z*−1; (v) −*x*, −*y*, *z*−1/2; (vi) *x*, *y*, *z*−1; (vii) −*x*, −*y*, *z*+1/2; (viii) −*x*, −*y*−1, *z*−1/2; (ix) −*x*+1/2, *y*+1/2, *z*−1/2; (x) *x*, *y*+1, *z*+1; (xi) −*x*, −*y*−1, *z*+1/2; (xii) −*x*+1/2, *y*+1/2, *z*+1/2; (xiii) *x*, *y*−1, *z*; (xiv) *x*, *y*+1, *z*.

### Hydrogen-bond geometry (Å, °)

**Table:**

*D*—H···*A*	*D*—H	H···*A*	*D*···*A*	*D*—H···*A*
O1—H1O···N1	0.82	1.88	2.602 (6)	147
N2—H2A···O3^vii^	0.80 (5)	2.30 (5)	3.076 (6)	163 (5)
C15—H15···O2^x^	0.93	2.49	3.259 (7)	140

Symmetry codes: (vii) −*x*, −*y*, *z*+1/2; (x) *x*, *y*+1, *z*+1.
